# Elevating SOX2 Downregulates MYC through a SOX2:MYC Signaling Axis and Induces a Slowly Cycling Proliferative State in Human Tumor Cells

**DOI:** 10.3390/cancers14081946

**Published:** 2022-04-12

**Authors:** Ethan P. Metz, Phillip J. Wilder, Tessa M. Popay, Jing Wang, Qi Liu, Achyuth Kalluchi, M. Jordan Rowley, William P. Tansey, Angie Rizzino

**Affiliations:** 1 Eppley Institute for Research in Cancer and Allied Diseases, Fred & Pamela Buffett Cancer Center, University of Nebraska Medical Center, Omaha, NE 68198, USA; ethan.metz@unmc.edu (E.P.M.); pwilder@unmc.edu (P.J.W.); 2 Department of Cell and Developmental Biology, Vanderbilt University School of Medicine, Nashville, TN 37232, USA; tessa.m.popay@vanderbilt.edu (T.M.P.); william.p.tansey@vanderbilt.edu (W.P.T.); 3 Department of Biostatistics, Vanderbilt University Medical Center, Nashville, TN 37232, USA; jing.wang.1@vumc.org (J.W.); qi.liu@vumc.org (Q.L.); 4 Center for Quantitative Sciences, Vanderbilt University Medical Center, Nashville, TN 37232, USA; 5 Department of Genetics, Cell Biology and Anatomy, University of Nebraska Medical Center, Omaha, NE 68198, USA; achyuth.kalluchi@unmc.edu (A.K.); jordan.rowley@unmc.edu (M.J.R.); 6 Department of Biochemistry, Vanderbilt University School of Medicine, Nashville, TN 37232, USA; 7 Department of Pathology and Microbiology, University of Nebraska Medical Center Fred & Pamela Buffett Cancer Center, Omaha, NE 68198, USA; 8 Department of Biochemistry and Molecular Biology, Fred & Pamela Buffett Cancer Center, University of Nebraska Medical Center, Omaha, NE 68198, USA

**Keywords:** SOX2, MYC (c-MYC), medulloblastoma, prostate cancer, colorectal cancer

## Abstract

**Simple Summary:**

The ability of slowly cycling/infrequently proliferating tumor cells to evade treatment with therapies that target actively proliferating tumor cells represents a major clinical challenge. Previous studies established that high levels of SOX2 in both fetal and tumor cells restrict cell proliferation and induce a slowly cycling state. However, the mechanisms through which elevated SOX2 levels inhibit tumor cell proliferation have not been identified. The studies presented here set out to determine the mechanisms through which SOX2 elevation restricts tumor cell proliferation. We demonstrated that elevating SOX2 decreases the expression of MYC and MYC target genes. We also determined that the downregulation of MYC is a critical mechanistic step necessary to maintain survival in the slowly cycling state induced by elevated SOX2. Altogether, our studies uncover a novel SOX2:MYC signaling axis and provide important insights into the molecular mechanisms through which SOX2 elevation induces a slowly cycling proliferative state.

**Abstract:**

Slowly cycling/infrequently proliferating tumor cells present a clinical challenge due to their ability to evade treatment. Previous studies established that high levels of SOX2 in both fetal and tumor cells restrict cell proliferation and induce a slowly cycling state. However, the mechanisms through which elevated SOX2 levels inhibit tumor cell proliferation have not been identified. To identify common mechanisms through which SOX2 elevation restricts tumor cell proliferation, we initially performed RNA-seq using two diverse tumor cell types. SOX2 elevation in both cell types downregulated MYC target genes. Consistent with these findings, elevating SOX2 in five cell lines representing three different human cancer types decreased MYC expression. Importantly, the expression of a dominant-negative MYC variant, omomyc, recapitulated many of the effects of SOX2 on proliferation, cell cycle, gene expression, and biosynthetic activity. We also demonstrated that rescuing MYC activity in the context of elevated SOX2 induces cell death, indicating that the downregulation of MYC is a critical mechanistic step necessary to maintain survival in the slowly cycling state induced by elevated SOX2. Altogether, our findings uncover a novel SOX2:MYC signaling axis and provide important insights into the molecular mechanisms through which SOX2 elevation induces a slowly cycling proliferative state.

## 1. Introduction

The stem cell transcription factor SOX2 plays diverse and critical roles during mammalian development and cancer. SOX2 has been implicated in the development and progression of more than 20 human cancer types [1]. In most of these cancers, SOX2 expression is correlated with negative outcomes. During mammalian development, SOX2 is required at many stages, including during peri-implantation development when SOX2-null embryos cease development [2]. Importantly, the effects of SOX2 across multiple contexts are highly dependent on its dosage [3]. Previous studies have demonstrated that although SOX2 is essential in embryonic stem cells [4], increasing SOX2 levels in these cells as little as twofold disrupts their self-renewal capacity and triggers differentiation [5]. Similarly, elevated levels of SOX2 relative to Oct4, Klf4, and c-MYC decrease the efficiency of reprogramming into induced pluripotent stem cells (iPSCs) [6,7,8].

SOX2 dosage is also important in governing its functions during development. In the developing nervous system, high levels of SOX2 maintain the stemness and quiescence of radial glial progenitor cells. These studies further demonstrated that the repression of SOX2 by proneural transcription factors was a critical regulatory step that enabled the proliferation and differentiation of neural progenitor cells [9]. Additional studies have shown that cells expressing high levels of SOX2 in endoderm-derived organs, such as the lung and stomach, are less proliferative relative to cells with lower levels of SOX2 expression. These studies also established a causal role between high levels of SOX2 and growth inhibition: interfering with SOX2 in the SOX2^high^ cells increased proliferation, whereas elevating SOX2 in their SOX2^low^ counterparts decreased proliferation [10].

Consistent with the growth inhibitory effects of SOX2 during development, high levels of SOX2 expression within a tumor are associated with slow cycling/infrequently proliferating cancer stem cells. In a mouse model of sonic hedgehog (SHH) medulloblastoma, high SOX2 expression is present in a small subpopulation of cells that proliferate infrequently. These slowly cycling SOX2^high^ cells exhibit resistance to chemotherapy and are able to repopulate the tumor and drive relapse [11]. High levels of SOX2 have also been observed in lung and colorectal tumor cells that display higher tumor-initiating capacities and decreased growth rates compared with SOX2^low^ cells within the tumor [12,13]. 

Slowly cycling/infrequently proliferating tumor cells present a significant clinical challenge because these cells demonstrate resistance to conventional chemotherapies that target rapidly proliferating cells. In animal models, these slow cycling tumor cells have been shown to provide a drug-resistant reservoir that can evade chemotherapy and drive tumor recurrence when chemotherapy is stopped [11]. In cancer xenograft models, treatment with several common chemotherapy regimens has been shown to induce a slowly cycling, drug-tolerant persister (DTP) cell state [14,15]. Significantly, the DTP cells exhibit phenotypic properties parallel to embryonic growth arrest, known as diapause. These properties include reversible growth inhibition, decreased MYC transcriptional activity, and reduced rates of protein biosynthesis [14,15,16,17,18]. Thus, tumor cells appear to hijack developmental growth arrest mechanisms enabling chemotherapy resistance. Understanding the mechanisms through which tumor cells induce the slowly cycling, drug-resistant state is critical to identifying therapeutic targets and enabling the eradication of these cells. 

Previous work from our laboratory has demonstrated that elevating SOX2 from a doxycycline (Dox)-inducible promoter inhibits the proliferation of multiple human cancer types [19,20,21,22]. For three of these tumor types, we have shown that SOX2 elevation in vivo induces a reversible state of tumor growth arrest, where tumor growth is abruptly halted when SOX2 is elevated until SOX2 returns to endogenous levels [22]. Intriguingly, growth inhibition by elevated SOX2 did not significantly disrupt the cell cycle distribution of engineered SOX2-inducible (i-SOX2) tumor cells. Rather, SOX2 elevation in these cells downregulated a broad spectrum of the cell cycle machinery governing progression through multiple phases of the cell cycle [22]. Altogether, our work and that of other groups strongly suggest that tumor cells hijack the growth inhibitory effects of elevated SOX2, consequently generating a reservoir of slowly cycling, drug-resistant tumor cells that can resist chemotherapy and drive disease recurrence. Given the significant clinical challenge posed by drug-resistant, slowly cycling tumor cells, understanding the molecular mechanisms through which elevated SOX2 induces this state should help identify therapeutic targets that will enable the eradication of these cells. The studies reported here set out to investigate the underlying mechanisms through which elevated SOX2 restricts tumor cell proliferation.

## 2. Materials and Methods

### 2.1. Cell Culture

ONS76, LNCaP, and DU145 and derived cell lines engineered for the inducible overexpression of SOX2: i-SOX2-ONS76, i-SOX2-LNCaP, and i-SOX2-DU145 have been described previously [21,22]. UW228 cells were obtained from Sutapa Ray (UNMC, Omaha, NE, USA). HCT116 cells were obtained from the American Type Culture Collection (Manassas, VA, USA). All cells, including i-SOX2-derived lines, were cultured in Dulbecco’s modified Eagle’s (DME) medium supplemented with 10% fetal bovine serum. Doxycycline (Dox, Clontech, Mountain View, CA, USA) was suspended in phosphate-buffered saline (PBS). SOX2 overexpression was induced by adding Dox to the culture medium at the times and concentrations indicated. Tamoxifen (4-OHT, Sigma, St. Louis, MO, USA) was dissolved in ethanol. MYC-ER nuclear translocation was induced by adding 4-OHT to the culture medium at the indicated times and concentrations. Cell proliferation was assessed by MTT assays performed on triplicate samples, as described previously [19,20,21,22]. Statistical significance was determined using a two-tailed Student’s *t*-test, with a significance threshold of *p* = 0.05. Cell lines not obtained from the American Type Culture Collection were verified by genetic analysis performed by the UNMC Molecular Diagnostics Laboratory. All cell lines were tested for the presence of mycoplasma and found to be negative. 

### 2.2. Vector Preparation and Construction

Cells were engineered for Dox-inducible SOX2 expression, as described previously [19,20,21,22]. pInducer-Omomyc has been previously described [23]. pLV-EF1a-cMyc-ER was created by insertion of the 2.3kB SalI-BamHI fragment containing the myc-ER coding sequence of pBabepuro-myc-ER (Addgene, Watertown, MA, USA, #19128) between the EcoRI and BamHI sites of pLV-EF1a-IRES-hygro (Addgene, Watertown, MA, USA, #85134). In this process, the SalI and EcoRI ends were blunted with Klenow before ligation. Lentiviral vectors were packaged as previously described [19,20,21,22]. Lentiviruses were diluted in DME + 10% FBS supplemented with 10 mM HEPES and 6 mg/mL polybrene and medium was added to cells. Successfully transduced cells were selected by puromycin, G418, or hygromycin resistance. 

### 2.3. RNA Isolation, cDNA Synthesis, and qPCR

RNA isolation was performed using the RNeasy kit (Qiagen, Germantown, MD. USA). Cells were lysed using RLT buffer and Qiashredder columns (Qiagen, Germantown, MD, USA), as outlined in the manufacturer’s protocols. RNA was then purified using RNeasy columns and eluted in RNase-free water. cDNA was synthesized using the Agilent High Fidelity 1st Strand cDNA synthesis kit (Agilent Technologies, La Jolla, CA, USA). cDNA was synthesized for 1 h at 42 °C, then terminated for 15 min at 70 °C. Primers used in qPCR to quantify mRNA are provided in Table 1. qPCR amplification and quantification were performed on a Bio-Rad CFX96 Real-Time PCR detection system (Bio-Rad, Hercules, CA, USA) and detected using RT^2^ SYBR Green qPCR mastermix (Qiagen, Germantown, MD, USA). Transcripts for each gene were measured in triplicate for each treatment group. Expression levels for target genes were confirmed using a second set of primers. *GAPDH* was used as a loading control. Statistical significance was determined using a two-tailed Student’s *t*-test with a significance threshold of *p* = 0.05. 

### 2.4. mRNA Sequencing

i-SOX2-ONS76, i-SOX2-LNCaP, and i-Omomyc-ONS76 cells were cultured in the presence or absence of Dox for 4 days. mRNA was harvested as outlined above. RNA samples were assessed for purity and quality. Library preparation and transcriptome sequencing on an Illumina NovaSeq 6000 platform were performed by Novogene (Beijing, China). For differential expression analysis, paired-end reads were aligned to the human genome version hg38 using STAR 2.7.3a guided by Ensembl gene annotations [24], and annotated transcripts were quantified and TPM normalized using Stringtie 2.1.1 [25]. Differential expression was assessed by DESeq2 [26] and significantly changed genes were required to have a Benjamini–Hochberg adjusted *p* value of < 0.05 and a 1.5-fold change in expression. Gene Ontology (GO) and gene set enrichment analysis (GSEA) were performed using WebGestalt [27] and GSEA, whereas P-HIPSTer pathogen–host predictions were obtained via Enrichr [28]. 

### 2.5. Chromatin Immunoprecipitation and Library Preparation 

Chromatin preparation was performed as described by Thomas et al. [29]. Confluent plates of ONS76 cells were cross-linked with 1% methanol-free formaldehyde (Thermo Fisher, Rockford, IL, USA, 8908) for 10 min before being quenched using 0.125mM glycine. Cells were then rinsed twice in ice-cold 1X PBS, harvested by scraping, and centrifuged. The resulting cell pellet was resuspended in formaldehyde lysis buffer (FALB: 50 mM HEPES pH 7.5, 140 mM NaCl, 1mM EDTA, and 1% Triton X-100) + 1% SDS + PIC (Roche, Denville, NJ, USA, 05056489001), and lysed on ice for 30 min. Cells were sonicated in a BioRuptor (Diagenode) for 25 min, 30s on/30s off, and debris removed by centrifugation. 

To chromatin prepared from ~10 million cells, 800 ng rabbit anti-IgG (Cell Signaling, Danvers, MA, USA, 2729S) or rabbit anti-MYC [Y69] (Abcam, Cambridge, UK, ab32072) was added, and samples were rotated overnight at 4 °C. Protein A Agarose (Roche, Denville, NJ, USA, 11134515001) was blocked with 10 μg BSA, added to each sample, and rotated at 4 °C for 2–4 h. Washes were then performed with low-salt wash buffer (20 mM Tris pH 8.0, 150 mM NaCl, 2 mM EDTA, 1% Triton X-100), high-salt wash buffer (20 mM Tris pH 8.0, 500 mM NaCl, 2 mM EDTA, 1% Triton X-100), lithium chloride wash buffer (10 mM Tris pH 8.0, 250 mM LiCl, 1 mM EDTA, 1% Triton X-100), and twice with TE (10 mM Tris pH 8.0, 1 mM EDTA). ChIP samples were de-crosslinked in 50 μL TE + 200 mM NaCl + 0.1% SDS + 20–40 μg PK (Macherey-Nagel, Duren, Germany, 740506) at 65 °C overnight. Three independent ChIP samples, performed using the same antibody with the same batch of chromatin, were combined. The DNA was then extracted with phenol–chloroform–isoamyl alcohol, and ethanol precipitated with glycogen (Roche, Denville, NJ, USA, 10901393001). 

Library preparation was performed using NEBNext Ultra II DNA Library Prep Kit for Illumina (NEB, Ipswich, MA, USA, E7645S) with dual indexing using NEBNext Multiplex Oligos for Illumina (NEB, Ipswich, MA, USA, E6440). An additional AMPure clean-up at the start of library preparation was included. Sequencing was carried out by Vanderbilt Technologies for Advanced Genomics (VANTAGE) using 150 bp paired-end sequencing on Illumina NovaSeq. 

### 2.6. Bioinformatics Analyses 

ChIP-Seq reads were aligned to the human genome hg19 using Bowtie2 [30]. Peaks in each sample were determined using MACS2 with q-value of 0.05 [31]. Consensus peaks in each condition were identified using DiffBind [32], where peaks occurring in at least two replicates were included. Peak locations were annotated using Homer [33] and assigned to putative nearby target genes. Enriched motifs were identified by the Homer command findMotifsGenome with the default region size and the motif length (-size 200 and -len 8, 10, 12). 

### 2.7. Western Blotting

Harvested cells were lysed using RIPA buffer (ThermoFisher, Rockford, IL, USA). RIPA lysis buffer was supplemented with protease and phosphatase inhibitors as previously described [34,35]. Western blot analysis was performed as previously described [34,35]. Nuclear and cytoplasmic protein extracts were prepared using the Pierce NE-PER^TM^ nuclear and cytoplasmic extraction kit (ThermoFisher, Rockford, IL, USA), as described previously [5,36]. The following antibodies from Cell Signaling Technology, Danvers, MA, USA, were used for Western blot analysis: SOX2 (#3579, 1:1000), MYC (#18583, 1:1000), cleaved Caspase-3 (#9664,1:1000), cleaved PARP (#5625,1:1000), LSD1 (#4218,1:1000), GAPDH (#2118,1:1000), HDAC1 (#34586, 1:1000), b-Tubulin (#2146, 1:1000). LSD1 and GAPDH were used as purity controls for nuclear and cytoplasmic fractions, respectively. HDAC1 and b-Tubulin were used as loading controls. Rabbit primary antibodies were detected with an anti-rabbit-IgG-AP secondary antibody (A3687, Sigma-Aldrich, St. Louis, MO, USA, 1:5000), as described previously [34,35]. Mouse primary antibodies were detected with an anti-mouse-IgG-AP secondary antibody (A4312, Sigma-Aldrich, 1:5000), as described previously. 

### 2.8. Cell Cycle Analysis

Cells were plated at subconfluent densities and cultured in the presence or absence of Dox for 4 days. Cells were then stained for cell cycle analysis according to the Telford method, as previously described [19]. Floating cells were collected and included in all cell cycle analyses. Flow cytometry analysis of triplicate samples was performed by the UNMC Flow Cytometry Research Facility.

### 2.9. Translation Assay

Nascent protein synthesis was measured following 48 h culture in the presence or absence of Dox using the Click-iT HPG Alexa Fluor 488 Protein Synthesis Assay Kit (ThermoFisher, Rockford, IL, USA), according to the manufacturer’s protocol. The fluorescent intensity of incorporated HPG-Alexa Fluor 488 in triplicate samples was measured by flow cytometry performed by the UNMC Flow Cytometry Research Facility. Mean fluorescence intensities for each treatment group were normalized to the mean fluorescent intensity of the control treatment group. Statistical significance was determined using a two-tailed Student’s *t*-test with a significance threshold of *p* = 0.05. 

### 2.10. Nuclear Run-On Assay 

Nuclear run-on was performed as described previously [37]. In brief, after 24 h of treatment with the indicated doses of Dox, cells were harvested via trypsin and lysed in NP-40 lysis buffer (10 mM Tris-HCl pH 7.4, 10 mM NaCl, 3 mM MgCl_2_, and 0.5% NP-40). Isolated nuclei were resuspended in nuclei storage buffer (50 mM Tris-HCl, pH 8.3, 0.1 mM EDTA, 5 mM MgCl_2_, 40% glycerol) and then mixed with an equal volume of 2× transcription reaction buffer (20 mM Tris-HCl, pH 8.3, 5 mM NaCl, 3 mM MgCl_2_, 300 mM KCl, 4 mM DTT) supplemented with 50 units RNase inhibitor, 1mM ATP, 1 mM GTP, 1 mM CTP, 0.5 mM UTP, and 0.5 mM 5-BrUTP (Sigma Aldrich, St. Louis, MO, USA). The reaction was incubated for 30 min at 30 °C. RNA from transcription was isolated with the MegaClear transcription clean-up kit, according to the manufacturer’s protocol. Purified RNA was incubated with 2 mg anti-BrdU antibody for 30 min at room temperature and then immunoprecipitated with Dynabeads Protein G (Invitrogen, Waltham, MA, USA). Precipitated RNAs were extracted by Trizol reagent (ThermoFisher, Rockford, IL, USA, 15596026).

## 3. Results

### 3.1. Elevating SOX2 Downregulates MYC Target Gene Expression

**Figure 1:**
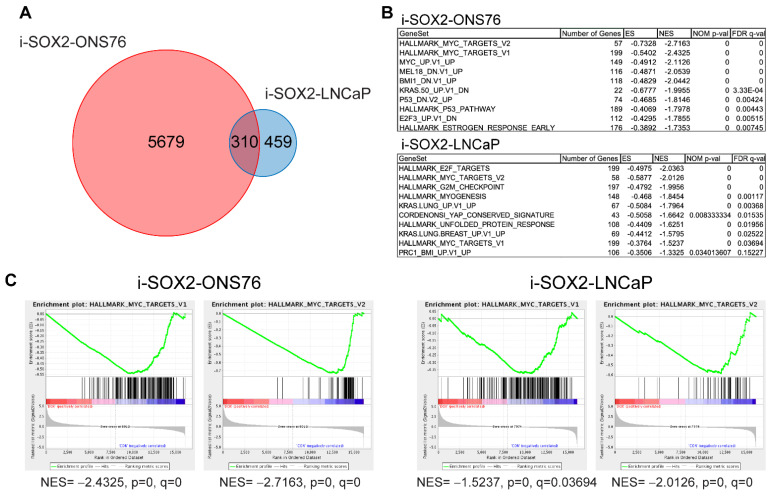
RNA-seq analysis of i-SOX2-ONS76 and i-SOX2-LNCaP cells. (**A**) Venn diagram of differentially expressed genes (DEGs) between i-SOX2-ONS76 and i-SOX2-LNCaP cells. (**B**) Top 10 downregulated GSEA gene sets when SOX2 is elevated in i-SOX2-ONS76 and i-SOX2-LNCaP cells. (**C**) Gene set enrichment plots for MYC target genes in i-SOX2-ONS76 and i-SOX2-LNCaP.

**Figure 2:**
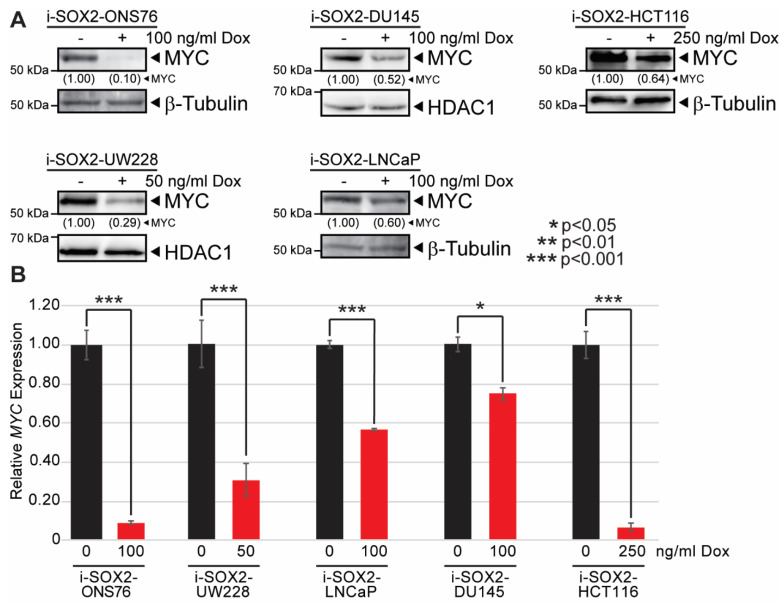
Elevating SOX2 downregulates MYC protein and mRNA in multiple tumor types. (**A**) Western blot analysis of MYC protein levels in i-SOX2 tumor cell lines after 48 h culture in the presence or absence of Dox at the indicated concentrations. (**B**) RT-qPCR analysis of MYC mRNA expression in i-SOX2 tumor cell lines after 48 h culture in the presence or absence of Dox at the indicated concentrations. Error bars represent standard deviation. * *p* < 0.05, *** *p* < 0.001.

Previous studies from our laboratory have shown that the inducible elevation of SOX2 reversibly inhibits the proliferation and tumor growth of multiple cancer cell types [19,20,21,22]. To identify potential mechanisms driving the growth-inhibitory effects of elevated SOX2, we performed RNA-seq using two diverse tumor cell types to identify genes and pathways affected in common. For these studies, SOX2-inducible medulloblastoma (i-SOX2-ONS76) and prostate tumor (i-SOX2-LNcaP) cells were used. Previous work in our laboratory has shown that both cell lines are growth-inhibited when SOX2 is elevated from an inducible promoter [21,22]. RNA-seq analysis identified 5989 and 769 differentially expressed genes (DEGs) when SOX2 was elevated in the i-SOX2-ONS76 and i-SOX2 LNCaP cells, respectively (Figure 1A and Figure S1A). Of these DEGs, 310 were affected in common between the two cell lines (Figure 1A). Based on our findings presented below (Figure 2A), we believe that the difference in number of DEGs between i-SOX2-ONS76 and i-SOX2-LNCaP cells may be due to differences in the magnitude of MYC downregulation when SOX2 is elevated in these cells. Gene set enrichment analysis (GSEA) of DEGs against oncogenic signature and hallmark gene sets [38] revealed that SOX2 elevation in each cell line significantly downregulated the expression of MYC target gene sets (HALLMARK_MYC_TARGETS_V1 and HALLMARK_MYC_TARGETS_V2, Figure 1B,C). To extend these findings, we searched the ChIP Enrichment Analysis (ChEA) database and found that genes downregulated upon SOX2 elevation in i-SOX2-ONS76 and i-SOX2-LNCaP cells were significantly enriched for MYC consensus target genes [39], as well as MYC’s obligate binding partner, MAX [40,41] (Figure S1C,D). Additionally, Gene Ontology (GO) analysis found that downregulated genes in both cell lines also demonstrated significant enrichment for biological processes regulated by MYC transcriptional activity, including protein translation, ribosome biogenesis, and cell cycle regulation (Figure S1C,D) [42,43]. Together, these findings indicate that SOX2 elevation downregulates the expression of MYC target genes in two diverse human cancer types. 

### 3.2. SOX2 Elevation Decreases MYC Expression in Multiple Human Tumor Cell Types

Our finding that elevating SOX2 downregulates the expression of MYC target genes led us to test whether elevating SOX2 affected c-MYC (hereafter referred to as MYC) expression. RNA-seq analysis of i-SOX2-ONS76 and i-SOX2-LNCaP cells showed that both cell lines express *MYC*, whereas *MYCL* and *MYCN* were not expressed at significant levels. Based on this finding, we focused our studies on examining how elevated SOX2 affected MYC expression. For these studies, we used five engineered i-SOX2 tumor cell lines, which included two medulloblastoma cell lines (i-SOX2-ONS76, i-SOX2-UW228), two prostate tumor cell lines (i-SOX2-DU145, i-SOX2-LNCaP), and one colorectal cell line (i-SOX2-HCT116). Our previous studies have shown that i-SOX2-ONS76, i-SOX2-LNCaP, and i-SOX2-DU145 cell are growth-inhibited when SOX2 is elevated [21,22]. Dox treatment of i-SOX2-HCT116 and i-SOX2-UW228 cells also resulted in a dose-dependent increase in SOX2 expression and a corresponding decrease in proliferation (Figure S2A,B). Consistent with the effects of SOX2 elevation in other SOX2-inducible cell lines [22], no significant alterations in cell cycle distribution were observed in growth-inhibited i-SOX2-HCT116 or i-SOX2-UW228 cells (Figure S2C). As shown for other tumor cell lines [19,20,21,22], Dox did not affect the proliferation of the parental, non-engineered HCT116 or UW228 cells (Figure S2A). To determine whether elevating SOX2 affects MYC expression, we examined the effects of elevated SOX2 on MYC protein levels by using concentrations of Dox that were sufficient to induce significant growth inhibition. In the cases of i-SOX2-ONS76 cells and i-SOX2-LNCaP cells, we used the concentration of Dox used in the RNA-Seq studies. Consistent with the downregulation of MYC target genes in our RNA-seq analysis, Western blot analysis of the five i-SOX2 cell lines revealed that elevating SOX2 substantially decreased MYC protein levels (Figure 2A). RT-qPCR analysis of the same i-SOX2 tumor cell lines demonstrated that *MYC* mRNA levels were significantly downregulated when SOX2 was elevated (Figure 2B). These results suggest that the downregulation of MYC target genes following SOX2 elevation is largely due to the downregulation of MYC. 

### 3.3. Identifying MYC-Bound Genes in ONS76 Cells

**Figure 3:**
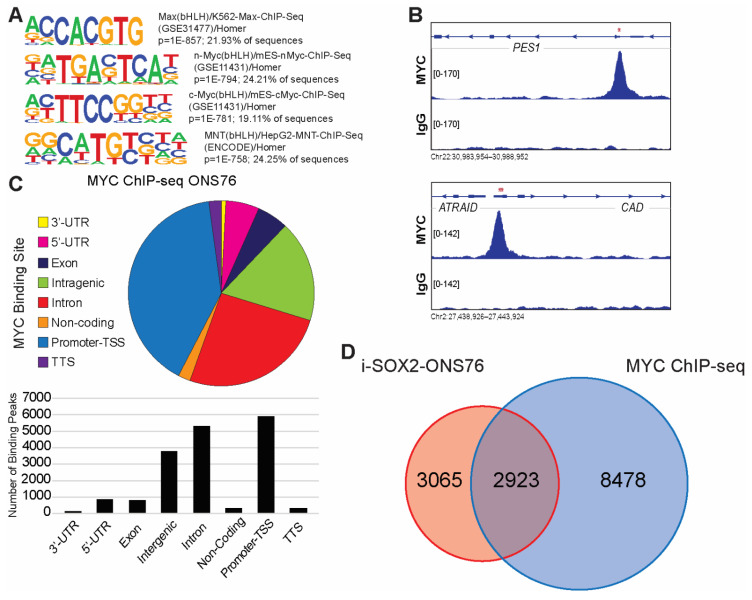
ChIP-seq analysis of MYC binding peaks in ONS76 cells. (**A**) Motif analysis for MYC-binding sites in ONS76 cells. Significance values and percentage of ChIP-seq peaks containing the motif are indicated. (**B**) IGV screenshot of representative ChIP-seq peaks for MYC in ONS76 cells. *PES1* contains an MYC-binding peak in a promoter/TSS region and *CAD* is bound near the TTS. Red asterisks indicate the position of E-boxes within the binding peaks. (**C**) Distribution of MYC binding peaks across regions of associated genes. (**D**) Venn diagram comparing i-SOX2-ONS76 DEGs and genes with associated MYC binding peaks.

The downregulation of hallmark MYC target gene sets and significant enrichment for MYC consensus target genes in genes downregulated following SOX2 elevation suggest that elevating SOX2 downregulates the expression of MYC target genes. To identify candidate MYC target genes, we performed chromatin immunoprecipitation sequencing (ChIP-seq) to map MYC binding sites in ONS76 cells. We did not perform ChIP-seq in the presence of elevated SOX2 in these cells due to the strong downregulation of MYC in these cells (Figure 2A). Binding peaks identified by our ChIP-seq analysis were significantly enriched in “E-box” consensus motifs (Figure 3A). MYC binding sites in ONS76 cells were dispersed across multiple gene regulatory elements (Figure 3B), with the most peaks mapping to promoter-TSS regions (Figure 3C). ChIP-seq analysis identified 17,350 MYC binding peaks associated with 11,401 genes in ONS76 cells, which is similar to MYC binding peaks observed in other cells [44]. GO analysis of the nearest genes to MYC binding peaks demonstrated significant enrichment for MYC-associated biological processes, including macromolecule biosynthesis, ribosome biogenesis, and transcription by RNA polymerase II [42,43] (Figure S3). Comparison of i-SOX2-ONS76 DEGs and genes bound by MYC identified by our ChIP-seq analysis revealed that 2923 of the 5989 DEGs (49%) in i-SOX2-ONS76 cells had associated MYC binding peaks (Figure 3D). Moreover, 2923 of the 11,401 (26%) of genes bound by MYC were differentially expressed following SOX2 elevation (Figure 3D). Thus, a large fraction of MYC-bound genes are differentially expressed when SOX2 is elevated in i-SOX2-ONS76 cells. 

### 3.4. Omomyc Induction Recapitulates the Growth Inhibitory Effects of Elevated SOX2

**Figure 4:**
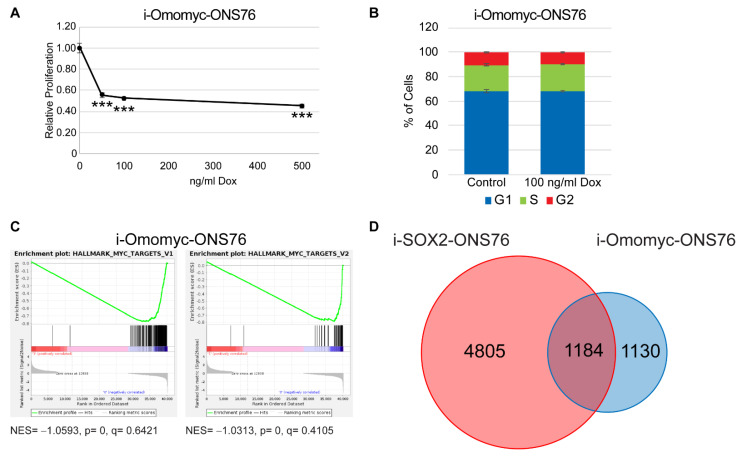
Omomyc induction recapitulates the proliferative and cell cycle effects of elevated SOX2. (**A**) Proliferation of i-Omomyc-ONS76 cells as determined by MTT assay following 4 days of culture in the presence of Dox at the indicated doses. (**B**) Cell cycle analysis of i-Omomyc-ONS76 cells was performed by flow cytometry after 4 days of culture in the presence or absence of 100 ng/mL Dox. (**C**) Gene set enrichment plots for MYC target genes in i-Omomyc-ONS76 cells. (**D**) Venn diagram comparing i-SOX2-ONS76 DEGs and i-Omomyc-ONS76 DEGs. Error bars represent the standard deviation. *** *p* < 0.001.

Although comparison of our i-SOX2-ONS76 RNA-seq and MYC ChIP-seq revealed substantial overlap between DEGs following SOX2 elevation and MYC-bound genes, it was not clear which MYC-bound genes were responsive to changes in MYC transcriptional activity. To identify MYC-regulated genes, we engineered ONS76 cells for the Dox-inducible expression of the dominant-negative MYC variant, omomyc. Omomyc consists of a region of the bHLH-Zip region of MYC with four point mutations introduced to enhance dimerization with MYC [45]. Treating i-Omomyc-ONS76 cells with Dox inhibited in vitro growth by approximately 50% following 4 days of Dox treatment (Figure 4A), similar to the extent of growth inhibition when SOX2 is elevated in i-SOX2-ONS76 cells [22]. Equally notable, cell cycle analysis of i-Omomyc-ONS76 cells revealed no significant changes in cell cycle distribution after 4 days of treatment (Figure 4B). The cell cycle effects of omomyc induction reflect our previous finding that SOX2 elevation does not significantly perturb the cell cycle distribution of SOX2-growth-inhibited cells [22]. Similar effects on proliferation and cell cycle were also observed in engineered i-Omomyc-HCT116 cells (Figure S4A,B). To identify MYC-responsive genes in ONS76 cells altered by omomyc, we performed RNA-seq analysis on i-Omomyc-ONS76 cells. RNA-seq identified 2314 DEGs when omomyc expression was induced (Figure 4D and Figure S5A). GSEA for oncogenic signatures and hallmark gene sets revealed that omomyc induction downregulated the same MYC target gene sets that were downregulated when SOX2 was elevated in ONS76 cells (Figure 4C). GO analysis of genes downregulated by omomyc demonstrated significant enrichment for MYC and MAX consensus target genes and MYC-associated biological processes including ribosome biogenesis and protein translation (Figure S5B) [42,43]. Comparison of i-Omomyc-ONS76 DEGs with MYC-bound genes identified by our ChIP-seq analysis revealed that 1006 (9%) of the 11,401 genes bound by MYC were differentially expressed following omomyc elevation (Figure S6). Significantly, 1184 (51%) DEGs in i-Omomyc-ONS76 cells were in common with the DEGs when SOX2 was elevated in i-SOX2-ONS76 cells (Figure 4D), demonstrating that SOX2 elevation significantly affected the expression of a large percentage of omomyc-induced DEGs. 

### 3.5. SOX2 and MYC Regulate Protein Translation-Associated Genes in ONS76 Cells

**Figure 5:**
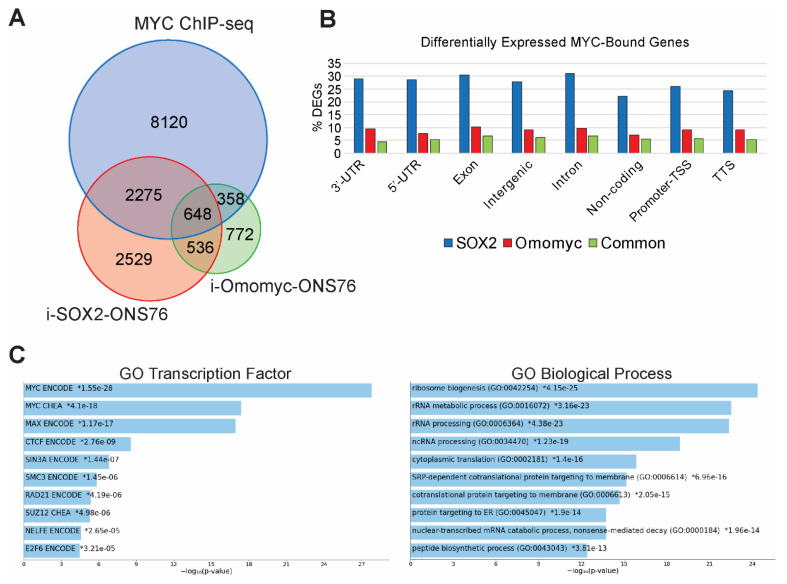
SOX2 and MYC regulate ribosomal biogenesis and protein translation genes in ONS76 cells. (**A**) Venn diagram showing the overlap of MYC-bound genes in ONS76 cells, i-SOX2-ONS76 DEGs, and i-Omomyc-ONS76 DEGs. (**B**) Percentage of MYC-bound genes differentially expressed in i-SOX2-ONS76, i-Omomyc-ON76, or both, based on the location of the MYC-binding peak. (**C**) TOP 10 GO Transcription Factor (ENCODE and CHEA) and Biological Process enrichment categories for common genes bound by MYC and differentially expressed in i-SOX2-ONS76 and i-Omomyc-ONS76 cells.

Comparison of MYC-bound genes identified by our ChIP-seq analysis of ONS76 cells with the DEGs identified by RNA-seq analysis of i-SOX2-ONS76 and i-Omomyc-ONS76 revealed that many genes associated with MYC binding peaks are in common with those altered when SOX2 or omomyc are elevated (Figure 5A,B). The parallel effects of SOX2 and omomyc elevation suggest a significant role for MYC target genes. Hence, we performed GO analysis on MYC-bound genes whose expression was altered in common when SOX2 and omomyc were elevated. As predicted, GO analysis of the genes that were bound by MYC in ONS76 and differentially expressed in both i-SOX2-ONS76 and i-Omomyc-ONS76 showed significant enrichment for genes containing MYC and MAX consensus motifs (Figure 5C). This analysis also revealed strong enrichment for biological processes related to protein translation, including ribosome biogenesis, translation, and rRNA metabolic process (Figure 5C and Figure S1C,D). This finding, together with the parallel effects of SOX2 and omomyc on proliferation, cell cycle, and gene expression, strongly suggests that the downregulation of MYC target genes plays a significant role in the growth inhibitory effects of elevated SOX2 by decreasing protein translation. 

### 3.6. SOX2 Elevation Decreases Protein Translation

**Figure 6:**
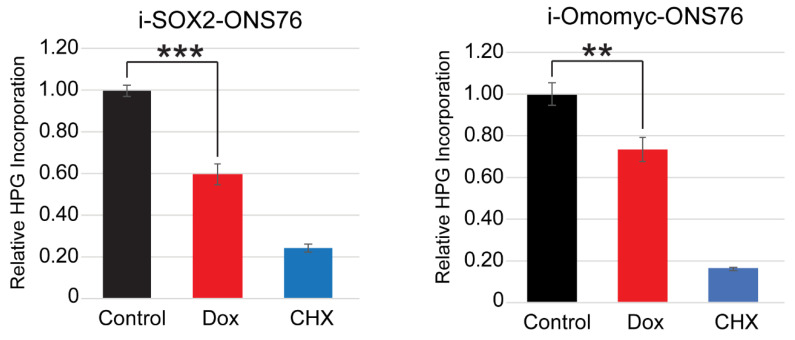
Elevating SOX2 and omomyc downregulates protein translation in ONS76 cells. Protein translation in i-SOX2-ONS76 and i-Omomyc-ONS76 cells was quantified by the flow cytometry analysis of HPG incorporation after 48 h culture in the presence of the indicated doses of Dox. Error bars represent standard deviations. ** *p* < 0.01, *** *p* < 0.001.

The downregulation of genes associated with protein translation in i-SOX2-ONS76, i-Omomyc-ONS76 and ONS76 ChIP-seq datasets led us to hypothesize that elevating SOX2 decreases protein translation. To test this hypothesis, i-SOX2 cells were treated for 2 days in the presence or absence of Dox, after which homopropargylglycine (HPG)-488 incorporation into nascent polypeptides was assessed as a measure of global protein translation rates. Elevating SOX2 in i-SOX2-ONS76 cells resulted in a 40% decrease in HPG incorporation when compared with the control treatment (Figure 6). Cycloheximide (CHX) was used as a positive control, and resulted in an approximately 80% decrease in HPG incorporation (Figure 6). Similar to elevating SOX2 in i-SOX2-ONS76 cells, omomyc induction significantly decreased HPG incorporation in i-Omomyc-ONS76 cells (Figure 6). Elevating SOX2 also significantly decreased HPG incorporation in i-SOX2-LNCaP and i-SOX2-HCT116 cells (Figure S7). Together, these results demonstrate that SOX2 elevation decreases protein translation across multiple human cancer types. Furthermore, the decreased protein translation when SOX2 is elevated may be a consequence of MYC downregulation, because omomyc elevation decreased protein translation to a similar extent as elevated SOX2 in ONS76 cells. 

### 3.7. MYC Rescue in the Presence of Elevated SOX2 Induces Cell Death

**Figure 7:**
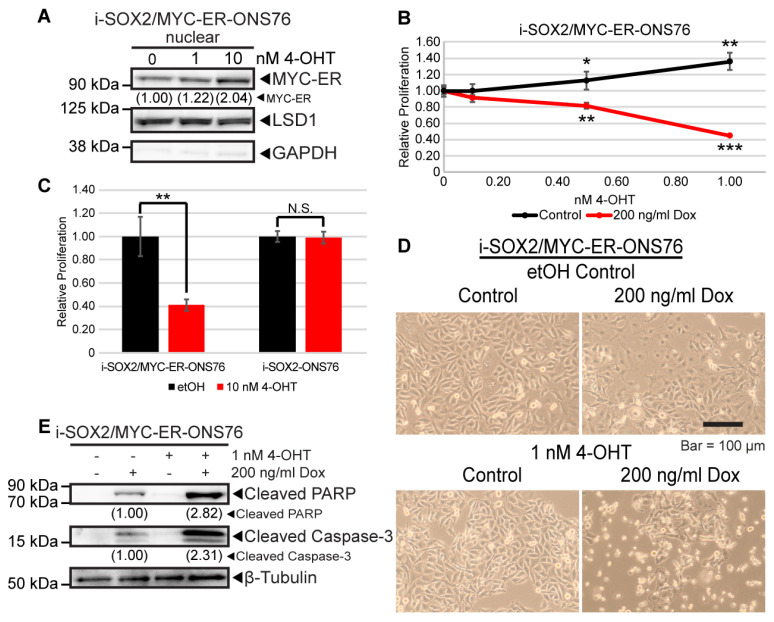
Elevated MYC activity in the context of elevated SOX2 induces cell death. (**A**) Western blot analysis of nuclear extracts from i-SOX2/MYC-ER-ONS76 cells. Extracts were harvested after 48 h in the presence or absence of Dox and 4-OHT. (**B**) Proliferation of i-SOX2/MYC-ER-ONS76 cells as determined by MTT assay following 4 days of culture in the presence of Dox and 4-OHT at the indicated doses. (**C**) Proliferation of i-SOX2/MYC-ER-ONS76 and i-SOX2-ONS76 cells was determined by MTT assay following 4 days of culture in the presence or absence of 10 nM 4-OHT. (**D**) Photomicrographs of i-SOX2/MYC-ER-ONS76 cells after 4 days of culture in the presence or absence of Dox and 4-OHT. (**E**) Western blot analysis of cleaved PARP and cleaved Caspase-3 using whole cell extracts prepared from cells treated for 48 h in the presence or absence of Dox and 4-OHT at the concentrations indicated. N.S., not significant; * *p* < 0.05, ** *p* < 0.01, *** *p* < 0.001.

Recapitulation of the effects of SOX2 on proliferation, cell cycle, and gene expression by omomyc strongly suggest that the downregulation of MYC downstream of elevated SOX2 plays an important role in the growth inhibitory effects of elevated SOX2. This led us to test whether rescuing the downregulation of MYC when SOX2 is elevated would reverse the growth inhibitory effects of elevated SOX2. To accomplish this, i-SOX2 tumor cell lines were engineered to constitutively express a tamoxifen (4-OHT)-inducible MYC-ER construct [46]. Treatment of i-SOX2/MYC-ER-ONS76 cells with 4-OHT increased the nuclear localization of MYC-ER (Figure 7A). Treating i-SOX2/MYC-ER-ONS76 cells with 1 nM 4-OHT for 4 days led to a small increase in cell proliferation (Figure 7B). Surprisingly, the same dose of 4-OHT in combination with Dox to elevate SOX2 significantly decreased proliferation (Figure 7B). Decreased proliferation was also observed at higher doses of 4-OHT (10 nM) in the absence of Dox (Figure 7C). Importantly, treatment with 10 nM 4-OHT did not affect the proliferation of i-SOX2-ONS76 cells, demonstrating that the effects observed are not due to the effects of 4-OHT on ONS76 cells. (Figure 7C). In contrast to the growth inhibitory effects of SOX2 elevation alone, which we have previously shown does not increase cell death [19,22], rescuing MYC activity in the presence of elevated SOX2 appeared to induce cell death. This was evident within 48 h by the appearance of floating cells (data not shown), which increased further after an additional 2 days (Figure 7D). For this study, we used a Dox concentration, which we had previously shown induces near-maximal growth inhibition after 4 day of Dox treatment [22]. Induction of cell death by elevated SOX2 and MYC-ER was confirmed by Western blot analysis, demonstrating PARP and Caspase-3 cleavage in Dox and 4-OHT-treated cells (Figure 7E). Low levels of PARP and Caspase-3 cleavage were also observed in Dox-treated cells not treated with 4-OHT, likely due to leaky MYC-ER present in the nucleus in the absence of tamoxifen (Figure 7A); our previous studies have shown that elevating SOX2 does not induce cell death [19,22]. Selective MYC-ER toxicity in the presence of elevated SOX2 was also observed in engineered i-SOX2/MYC-ER-HCT116 cells (Figure S8). Importantly, these findings demonstrate that rescuing MYC activity leads to cell death when SOX2 is elevated. Thus, the downregulation of MYC is critical for maintaining cell viability when growth is restricted by elevated SOX2. 

### 3.8. SOX2 Elevation Transcriptionally Downregulates MYC

**Figure 8:**
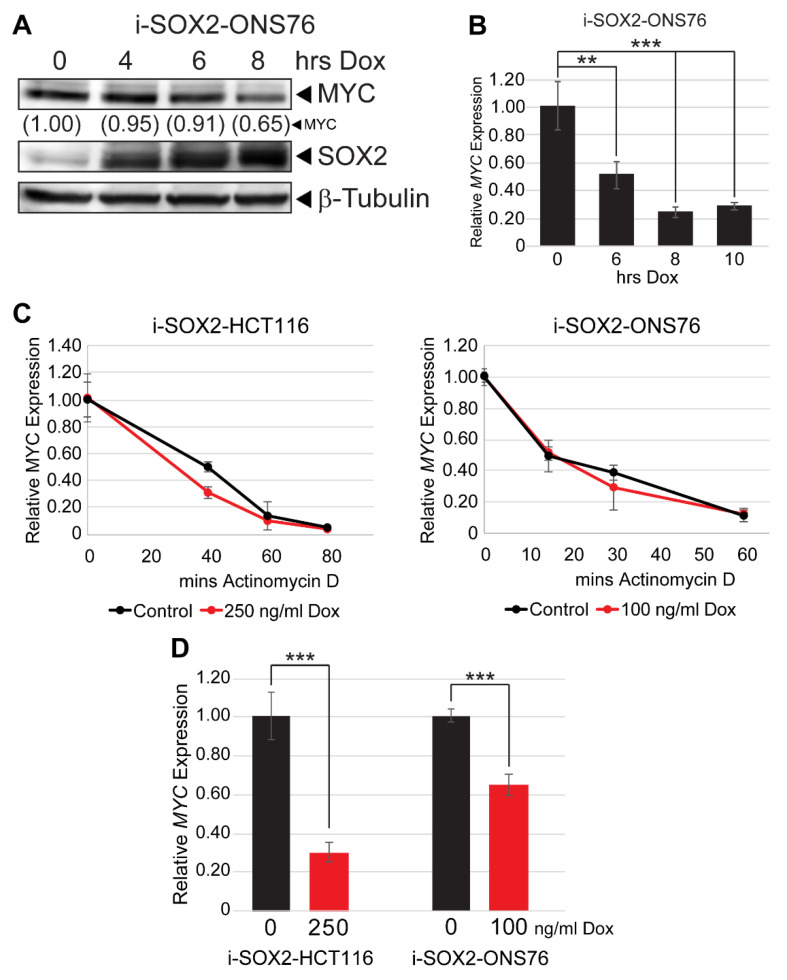
SOX2 elevation downregulates MYC transcription. (**A**) Western blot analysis of MYC protein levels in i-SOX2-ONS76 cells treated with 100 ng/mL Dox for the indicated times. (**B**) RT-qPCR analysis of *MYC* mRNA levels in i-SOX2-ONS76 cells treated with 100 ng/mL Dox for the indicated times. Error bars represent standard deviations. (**C**) RT-qPCR analysis of *MYC* mRNA levels after 24 h of treatment at the indicated Dox dosage followed by 5 mg/mL actinomycin D treatment for the times indicated. *MYC* mRNA levels in control and Dox-treated cells were normalized to the 0 actinomycin D time point. (**D**) NRO-qPCR analysis of i-SOX2-ONS76 and i-SOX2-HCT116 cells. Cells were cultured in the indicated doses of Dox for 24 h before being subjected to NRO-qPCR analysis. Error bars represent standard deviation. ** *p* < 0.01, *** *p* < 0.001.

Given the parallel effects of omomyc and SOX2, we set out to determine how SOX2 decreases MYC expression. Having previously demonstrated that MYC protein and mRNA were both downregulated when SOX2 was elevated, we performed a series of time-course studies to determine the relative kinetics of MYC mRNA and protein downregulation by elevated SOX2. Western blot analysis demonstrated that SOX2 was significantly elevated in ONS76 cells approximately 4 h after Dox was added to the culture media, whereas MYC protein was downregulated as early as 8 h after Dox was added (Figure 8A). Significantly, *MYC* mRNA was decreased nearly 50% at 6 h after Dox administration, with further downregulation observed at 8 and 10 h of treatment (Figure 8B). The downregulation of MYC mRNA prior to decreased protein levels led us to hypothesize that the MYC downregulation by elevated SOX2 is driven primarily by the downregulation of MYC at the RNA level. Consistent with this hypothesis, SOX2 elevation did not significantly decrease MYC protein half-life in i-SOX2-HCT116 or i-SOX2-ONS76 cells (Figure S9). To determine whether elevating SOX2 affected *MYC* mRNA half-life, i-SOX2-HCT116 and i-SOX2-ONS76 cells were treated for 24 h in the presence or absence of Dox, at which point the cells were treated with actinomycin D to inhibit transcription. RT-qPCR analysis demonstrated that SOX2 elevation did not significantly decrease *MYC* mRNA half-life in either i-SOX2-HCT116 or i-SOX2-ONS76 cells (Figure 8C). Finally, we tested whether elevating SOX2 decreased MYC at the transcriptional level. For this purpose, we performed nuclear run-on (NRO) qPCR after 24 h of treatment in the presence or absence of Dox. NRO-qPCR revealed that SOX2 elevation significantly decreased *MYC* transcription in i-SOX2-HCT116 and i-SOX2-ONS76 cells (Figure 8D). Altogether, these findings demonstrate that elevated SOX2 primarily downregulates MYC at the transcriptional level. 

## 4. Discussion

The findings presented in this study provide novel insights into the molecular mechanisms through which elevated SOX2 restricts the proliferation of multiple human cancer types. Specifically, we demonstrated that SOX2 elevation leads to the downregulation of MYC and MYC target genes. The downregulation of MYC target genes by elevated SOX2 was validated through multiple experiments. GSEA of our RNA-seq datasets identified HALLMARK_MYC_TARGETS_V1 and HALLMARK_MYC_TARGETS_V2 as commonly downregulated gene sets when SOX2 is elevated in i-SOX2-ONS76 and i-SOX2-LNCaP cells. Additionally, ChEA and GO analysis of genes downregulated when SOX2 is elevated in i-SOX2-ONS76 and i-SOX2-LNCaP revealed significant enrichment for MYC and MAX consensus target genes [39]. Comparison of the DEGs between i-SOX2-ONS76 and i-Omomyc-ONS76 cells showed that 51% of DEGs following omomyc elevation were commonly altered when SOX2 was elevated. Importantly, our MYC ChIP-seq analysis demonstrated that 49% of i-SOX2-ONS76 DEGs were bound by MYC. In addition to i-SOX2-LNCaP and i-SOX2-ONS76, characterization of three additional engineered i-SOX2 tumor cell lines indicated that elevating SOX2 decreases MYC mRNA and protein in five tumor cell lines representing three human cancer types (prostate, medulloblastoma, and colorectal). Thus, the downregulation of MYC is a common response when cell proliferation is inhibited by elevated SOX2. 

Our studies also demonstrate that the downregulation of MYC plays a critical role in the growth inhibitory effects of elevated SOX2. First, the expression of dominant-negative omomyc recapitulates many of the effects of elevated SOX2. Our previous work has shown that SOX2 elevation in five cell lines representing three tumor types (prostate, pancreatic, and medulloblastoma) does not significantly perturb the cell cycle distribution of engineered cells despite inhibiting growth [22]. Here, we have presented this cell cycle phenotype in two additional cell lines (HCT116 and UW228) and demonstrated that omomyc elevation does not significantly alter the cell cycle distribution of ONS76 and HCT116 cells. The recapitulation of this cell cycle phenotype suggests that omomyc and SOX2 inhibit proliferation through similar mechanisms. Second, our RNA-seq analyses of i-SOX2-ONS76 and i-Omomyc-ONS76 revealed significant overlap in DEGs when SOX2 and omomyc are elevated. A total of 1184 genes were commonly affected between these two datasets, representing 20% and 51% of DEGs in the i-SOX2-ONS76 and i-Omomyc-ONS76, respectively. Third, we demonstrate that rescuing the downregulation of MYC activity by elevated SOX2 using a tamoxifen-inducible MYC-ER induces cell death. In contrast, we have previously shown that elevating SOX2 does not induce cell death [19,22]. Thus, the downregulation of MYC by elevated SOX2 is an essential mechanism to maintain the viability of SOX2-growth-inhibited cells. 

The findings presented here demonstrate that elevating SOX2 primarily downregulates MYC at the transcriptional level. We observed little change in the turnover of MYC protein or MYC RNA when SOX2 was elevated. Currently, it is unclear how elevated SOX2 downregulates MYC transcription. However, GO analysis of genes upregulated by elevated SOX2 in i-SOX2-ONS76 and i-SOX2-LNCaP cells identified genes reported to negatively regulate Wnt signaling (Figure S10). This is interesting, because β-catenin has been implicated in the expression of the MYC [47]. However, thus far, we have not observed any decreases in the expression of β-catenin or its nuclear localization when SOX2 is elevated (data not shown). Given the importance of MYC downregulation in SOX2-induced growth inhibition, it will be important to investigate the molecular mechanism(s) by which elevated SOX2 interferes with MYC expression.

Together with previous work from our laboratory [19,20,21,22], the findings presented here demonstrate that elevating SOX2 in seven types of human cancer invariably leads to growth inhibition, strongly suggesting that this is a general effect of elevated SOX2 in human cancer. Our finding that elevated SOX2 restricts tumor cell proliferation reflects the effects of high SOX2 expression during development and cancer [3]. Several previous studies have demonstrated that cells expressing high levels of SOX2 in the developing nervous system, stomach, esophagus, and lung exhibit decreased proliferation compared with cells expressing lower SOX2 levels [9,10]. Importantly, these studies demonstrated a causal relationship between elevated SOX2 and growth inhibition because the expression of a dominant negative form of SOX2 in SOX2^high^ cells increased their proliferation [10]. A similar relationship between high SOX2 expression and limited proliferation has been demonstrated in the auditory epithelium. Inner pillar cells within the auditory epithelium express high levels of SOX2 and the tumor suppressor p27^Kip1^. Significantly, SOX2 knockdown was sufficient to decrease the expression of p27^Kip1^ and increase proliferation in these cells [48]. 

Our finding that elevated SOX2 restricts the proliferation of tumor cells is also consistent with the reports of high SOX2 expression in infrequently proliferating tumor cells. SOX2 expression in mouse models of SHH medulloblastoma has been demonstrated to be restricted to a subpopulation consisting of approximately 5% of tumor cells that exist in a slowly cycling state. These SOX2^high^ cells exhibited resistance to chemotherapy, and lineage tracing studies revealed that recurrent tumors were derived from the SOX2^high^ cells [11]. Another report demonstrated that the intracardiac injection of lung tumor cells disseminated to multiple sites and remained dormant for up to 240 days. Characterization of the disseminated cells demonstrated that the cells expressed elevated levels of SOX2 compared with the parental cell population and were enriched for tumor-initiating cells [12]. Together, these studies strongly suggest that slow cycling/infrequently proliferating tumor cells hijack the growth restricting effects of elevated SOX2 operative during development resulting in chemotherapy resistance.

Studies described in this report, together with our previous work [19,20,21,22], demonstrate that elevating SOX2 above endogenous levels leads to growth inhibition in multiple tumor cell lines representing different tumor types. This is true for cells that express low levels of SOX2, such as LNCaP prostate tumor cells, or higher levels of SOX2, such as ONS76 medulloblastoma cells [21,22]. However, this does not mean that SOX2 levels do not rise during tumor progression. There is ample evidence that SOX2 levels rise during tumor progression in some cancers [1]. As discussed elsewhere [1,2], we have argued that increases in the levels of SOX2 can contribute to tumor growth, provided the increases in SOX2 are accompanied by changes in the expression of other genes that are able to counter the growth inhibitory effects of increased SOX2. For example, mutations in two tumor suppressor genes, RB1 and TP53, that are observed in high-risk prostate cancer patients, lead to large increases in SOX2 without blocking tumor growth [49,50]. Thus far, the changes in genes that enable SOX2 levels to rise in different cancers without blocking tumor growth have not been studied adequately. We speculate that the genes needed to counterbalance the inhibitory effects of elevated SOX2 are likely to vary by cancer type. Similarly, it is likely that the stage at which SOX2 levels rise during tumor progression is likely to vary by cancer type. For example, in the case of prostate cancer, SOX2 levels rise in concert with increases in Gleason score [51], whereas in other cancers, such as SHH medulloblastoma, it is unclear whether SOX2 levels rise during tumor progression. For these tumors, SOX2 is expressed in their likely cell of origin [52,53]. 

## 5. Conclusions

The findings presented here uncover a novel SOX2:MYC signaling axis that regulates tumor cell proliferation and demonstrate that SOX2 elevation decreases the expression of MYC and downregulates MYC target genes. We show that this signaling axis is operative across different types of human cancer and may affect additional tumor contexts given that SOX2 is expressed and implicated in at least 20 types of human cancer [1] and MYC is deregulated in up to 70% of cancers [42].

## Figures and Tables

**Table 1:** Primers used for qPCR.

Name	Forward Primer Sequence 5′-3′	Reverse Primer Sequence 5′-3′
GAPDH	ACAGCGACACCCACTCCTCC	GAGGTCCACCACCCTGTTGC
MYC-1	GGCTCCTGGCAAAAGGTCA	CTGCGTAGTTGTGCTGATGT
MYC-2	GTCAAGAGGCGAACACACAAC	TTGGACGGACAGGATGTATGC

## Data Availability

RNA-seq data have been deposited at the Gene Expression Omnibus (GEO; GSE186834) with a link for reviewers to access at: https://www.ncbi.nlm.nih.gov/geo/query/acc.cgi?acc=GSE190936 [54] (accessed on 8 December 2021) using the secure token “ufyjaueextybvcx”. ChIP-seq data have been deposited at the Gene Expression Omnibus (GEO; GSE186834) with a link for reviewers to access at: https://www.ncbi.nlm.nih.gov/geo/query/acc.cgi?acc=GSE186834 [55] (accessed on 9 November 2021) using the secure token “yrsbksoovrmfpgr”.

## Supplementary Materials

The following supporting information can be downloaded at: https://www.mdpi.com/article/10.3390/cancers14081946/s1, Figure S1: GO analysis of genes downregulated in i-SOX2-ONS76 and i-SOX2-LNCaP cells; Figure S2: Effects of SOX2 elevation on i-SOX2-HCT116 and i-SOX2-UW228 cells; Figure S3: Gene ontology analysis of MYC-bound genes identified by ChIP-seq in ONS76 cells; Figure S4: Effects of omomyc induction on the proliferation and cell cycle of i-Omomyc-HCT116 cells; Figure S5: RNA-seq analysis of i-Omomyc-ONS76 cells; Figure S6: Comparison of i-Omomyc-ONS76 DEGs and MYC-bound genes in ONS76 cells; Figure S7: Elevating SOX2 decreases protein translation in i-SOX2-LNCaP and i-SOX2-HCT116 cells; Figure S8: Effects of MYC-ER induction on the proliferation of i-SOX2/MYC-ER-HCT116 cells; Figure S9: SOX2 elevation does not significantly affect MYC protein half-life; Figure S10: Gene ontology analysis of genes upregulated by elevated SOX2 in i-SOX2-ONS76 and i-SOX2-LNCaP cells.
