# Supplementary material for: Elevating SOX2 Downregulates MYC through a SOX2:MYC Signaling Axis and Induces a Slowly Cycling Proliferative State in Human Tumor Cells
Source: Cancers (Basel). 2022 Apr 12;14(8):1946. doi: 10.3390/cancers14081946 (PMC9025961; doi:10.3390/cancers14081946)
Supplement: Supplementary file 1 [file cancers-14-01946-s001.zip › cancers-1658888-supplementary.pdf]

Supplemental Figure Legends

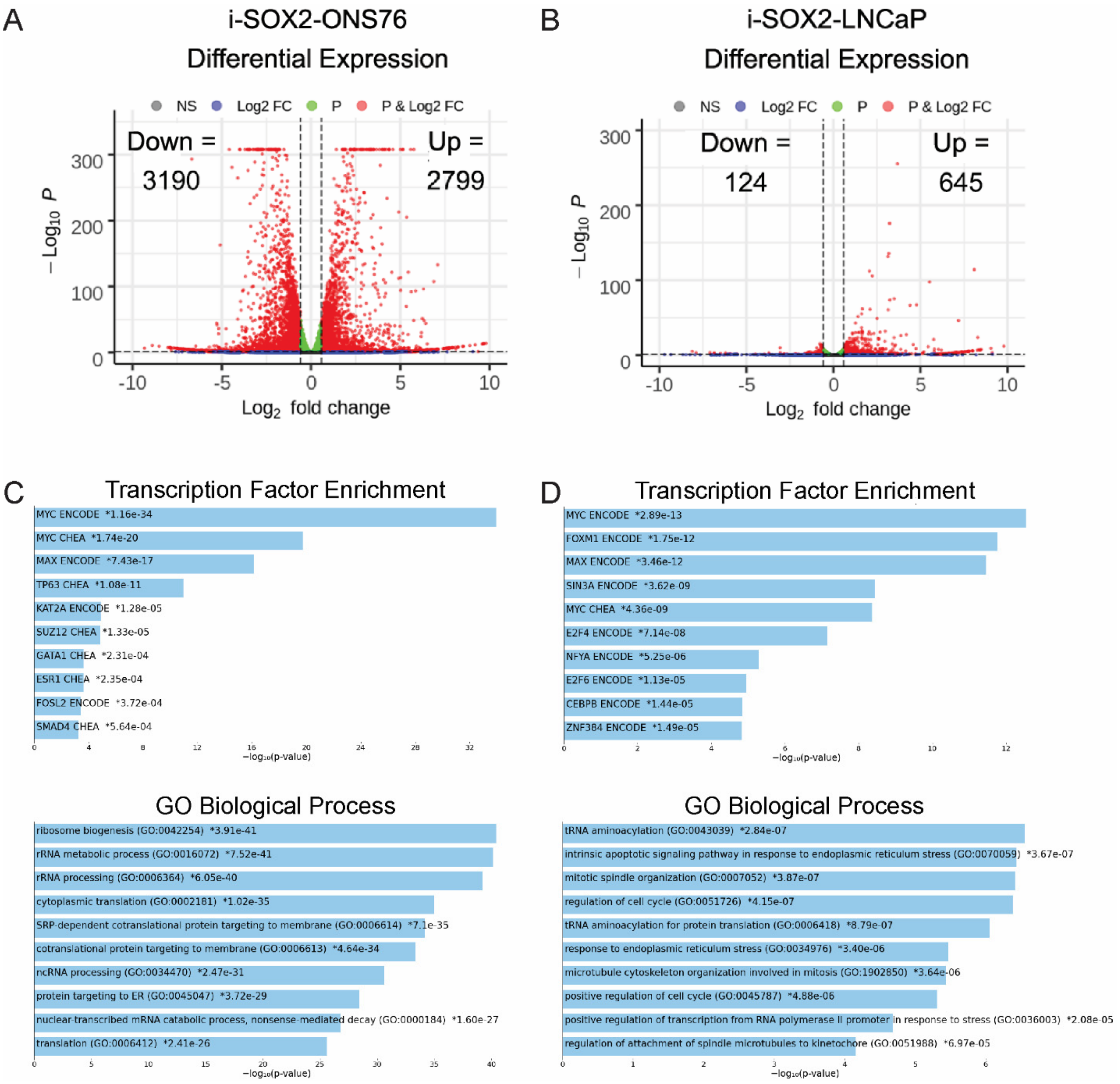

Figure S1. GO analysis of genes downregulated in i-SOX2-ONS76 and i-SOX2-LNCaP cells. Volcano plots of differentially expressed genes in **A.** i-SOX2-ONS76 and **B.** i-SOX2-LNCaP cells. Gene ontology enrichment for transcription factor binding sites (ENCODE and ChEA Consensus TF from ChIP-X) and GO Biological Process for genes downregulated in **C.** i-SOX2-ONS76 and **D.** i-SOX2-LNCaP DEGs.

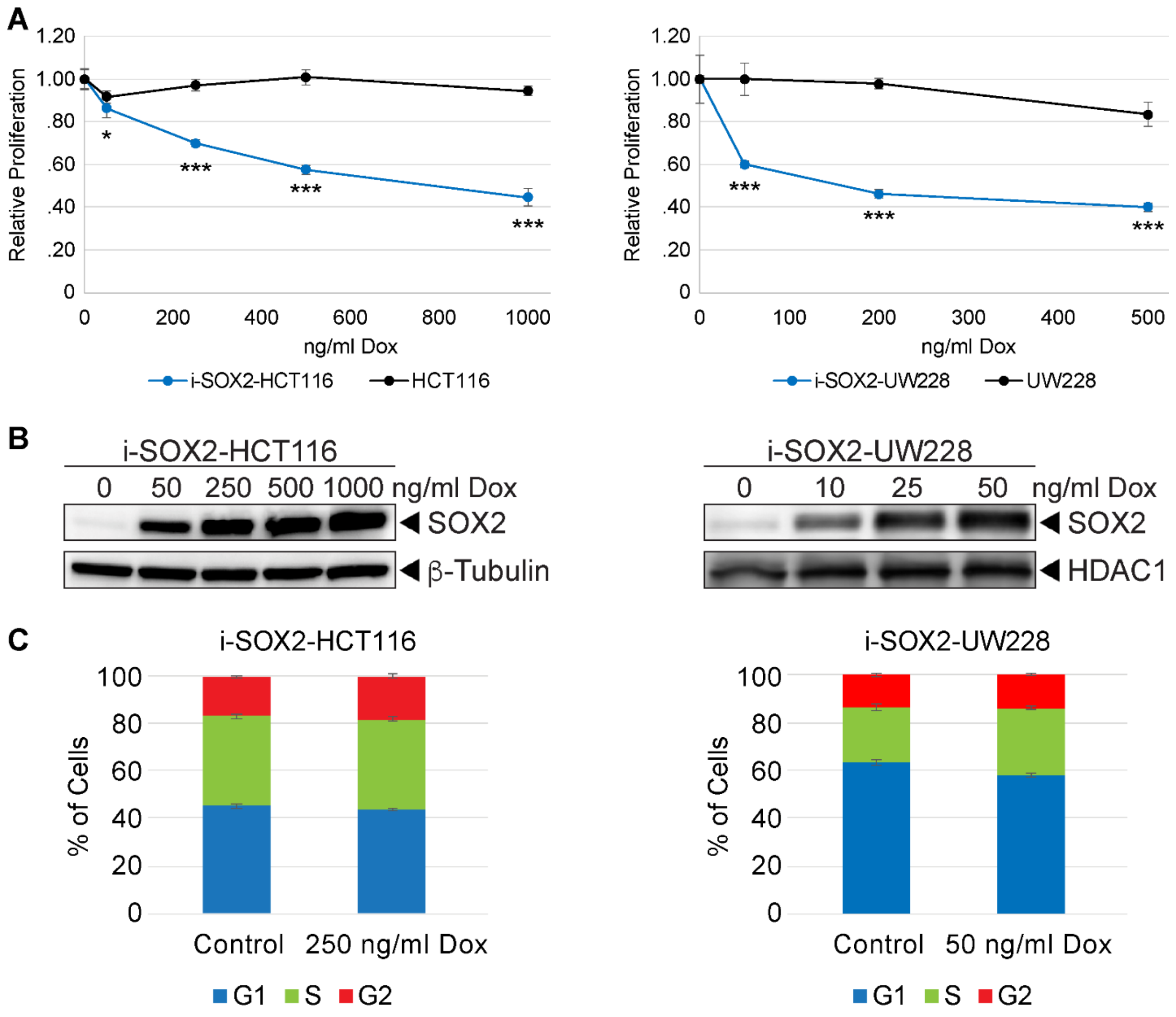

Figure S2. Effects of SOX2 elevation on i-SOX2-HCT116 and i-SOX2-UW228 cells. A. Proliferation of HCT116, i-SOX2-HCT116, UW228, and i-SOX2-UW228 cells as determined by MTT assays after 4 days culture in the presence of the indicated doses of Dox. **B.** Western blot analysis of whole cell extracts from i-SOX2-HCT116 and i-SOX2-UW228 cultured for 48 hours in the indicated doses of Dox. **C.** Cell cycle analysis of i-SOX2-HCT116 and i-SOX2-UW228 cells assessed by flow cytometry following 4 days culture in the presence of absence of Dox at the doses indicated. Error bars represent standard deviation.

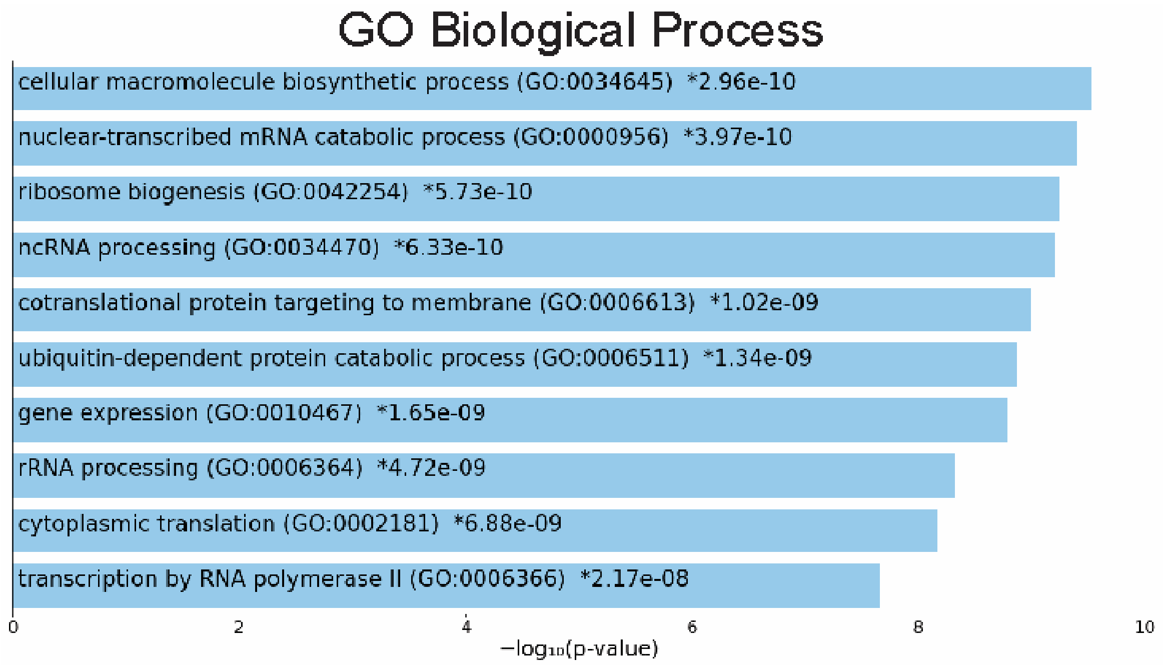

Figure S3. Gene ontology analysis of MYC bound genes identified by ChIP-seq in ONS76 cells. Top 10 GO Biological Process enrichment categories for MYC-bound genes in ONS76 cells.

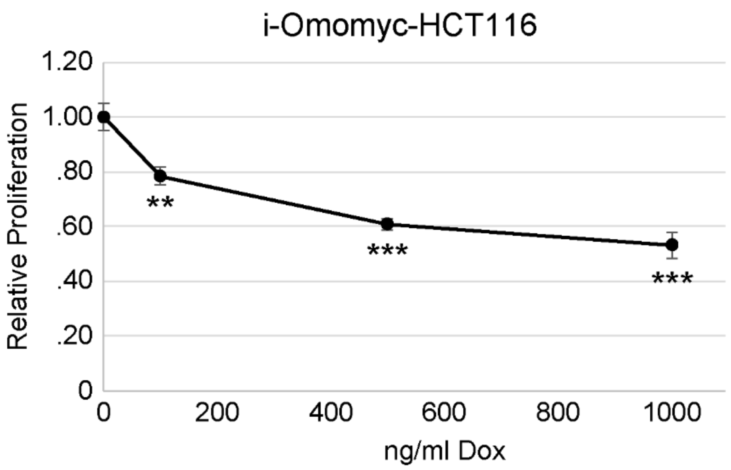

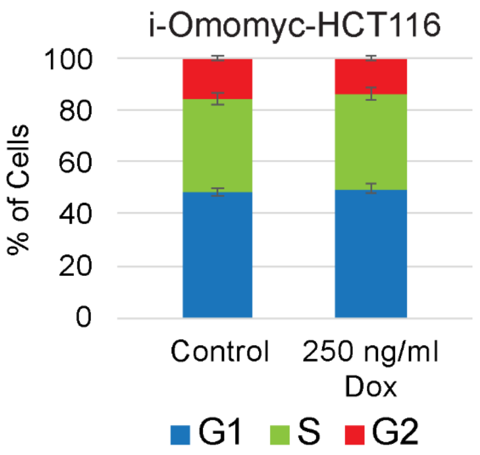

Figure S4. Effects of omomyc induction on the proliferation and cell cycle of i-Omomyc-HCT116 cells. A. Proliferation of i-Omomyc-HCT116 cells was determined by MTT assay after 4 days culture in the presence of Dox at the indicated doses. **B.** Cell cycle analysis of i-Omomyc-HCT116 cells measured by flow cytometry analysis after 4 days culture in the presence or absence of 250 ng/ml Dox.

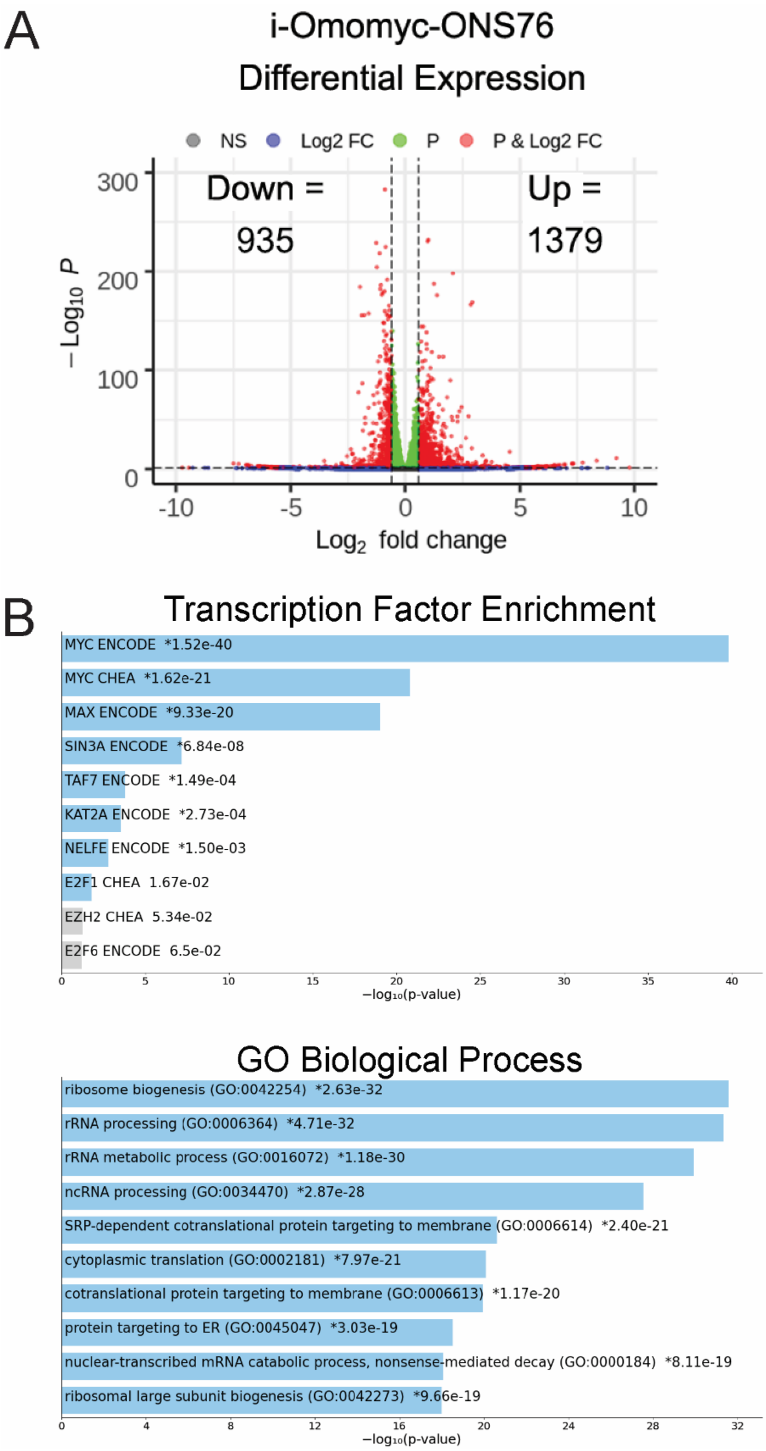

Figure S5. RNA-seq analysis of i-Omomyc-ONS76 cells. A. Volcano plot of differentially expressed genes following Dox treatment of i-Omomyc-ONS76 cells. **B.** TOP 10 GO Transcription Factor (ENCODE and CHEA) and Biological Process enrichment categories for genes downregulated following omomyc elevation in i-Omomyc-ONS76 cells.

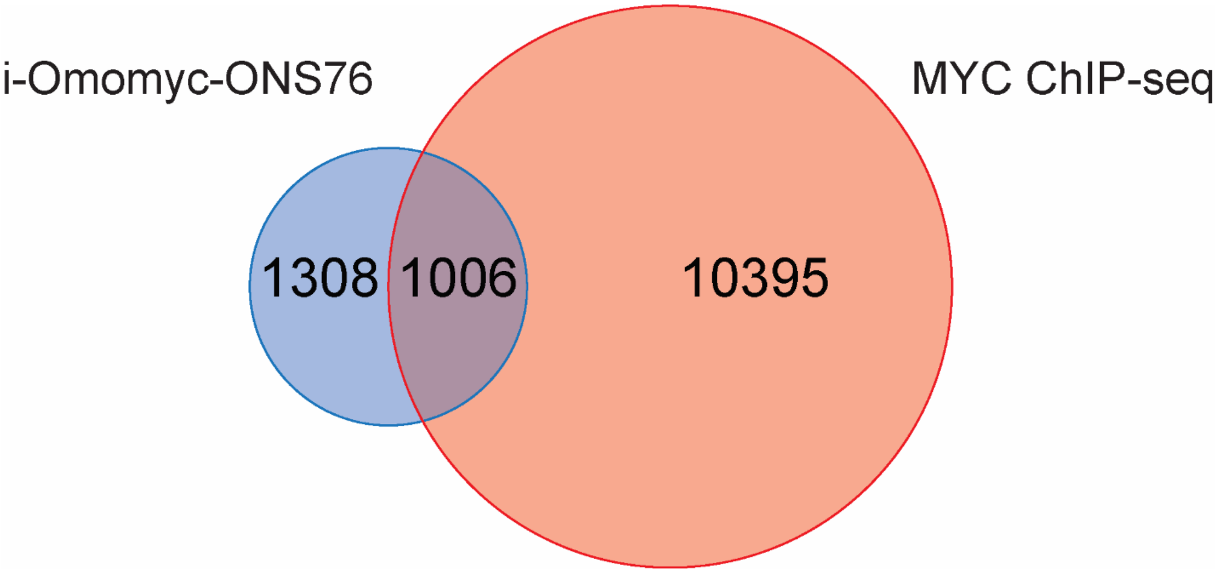

Figure S6. Comparison of i-Omomyc-ONS76 DEGs and MYC-bound genes in ONS76 cells. Venn diagram showing overlap of MYC-bound genes in ONS76 cells and i-Omomyc-ONS76 DEGs.

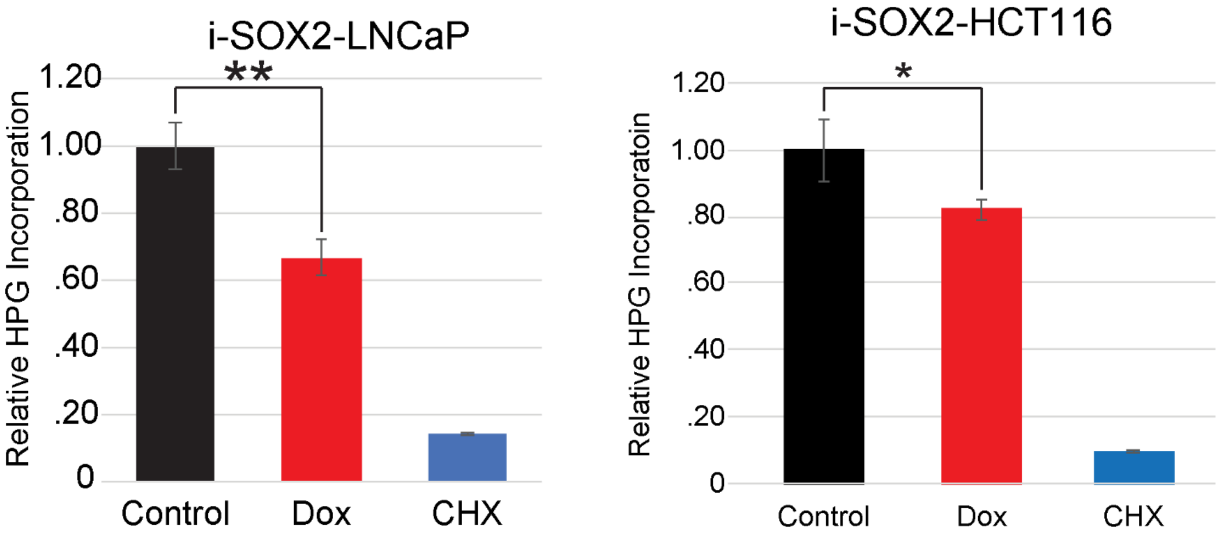

Figure S7. Elevating SOX2 decreases protein translation in i-SOX2-LNCaP and i-SOX2-HCT116 cells. Protein translation in i-SOX2-LNCaP and i-SOX2-HCT116 cells was quantified by

flow cytometry analysis of HPG incorporation after 48 hrs culture in the presence of the indicated doses of Dox. Error bars represent standard deviation.

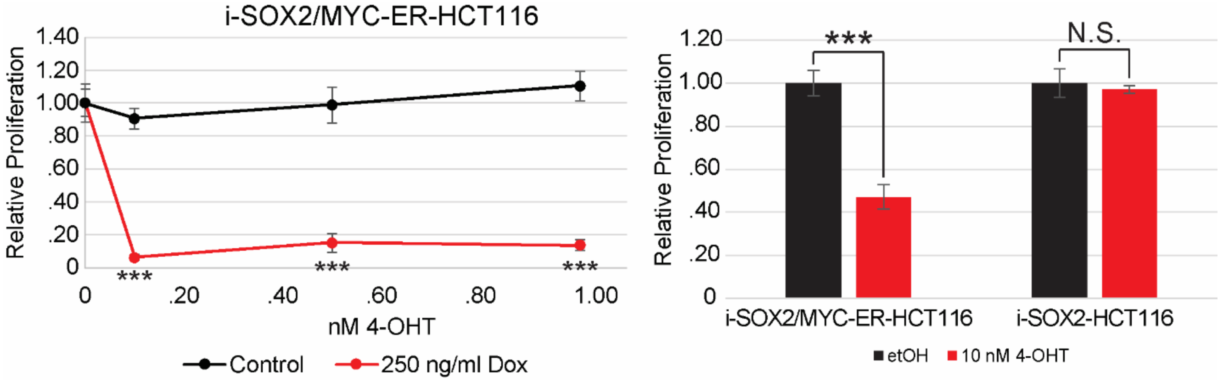

Figure S8. Effects of MYC-ER induction on the proliferation of i-SOX2/MYC-ER-HCT116 cells. A. Proliferation of i-SOX2/MYC-ER-HCT116 cells as determined by MTT assay following 4 days culture in the presence of Dox and 4-OHT at the indicated doses. **B.** Proliferation of i-SOX2/MYC-ER-HCT116 and i-SOX2-HCT116 cells was determined by MTT assay following 4 days culture in the presence or absence of 10 nM 4-OHT.

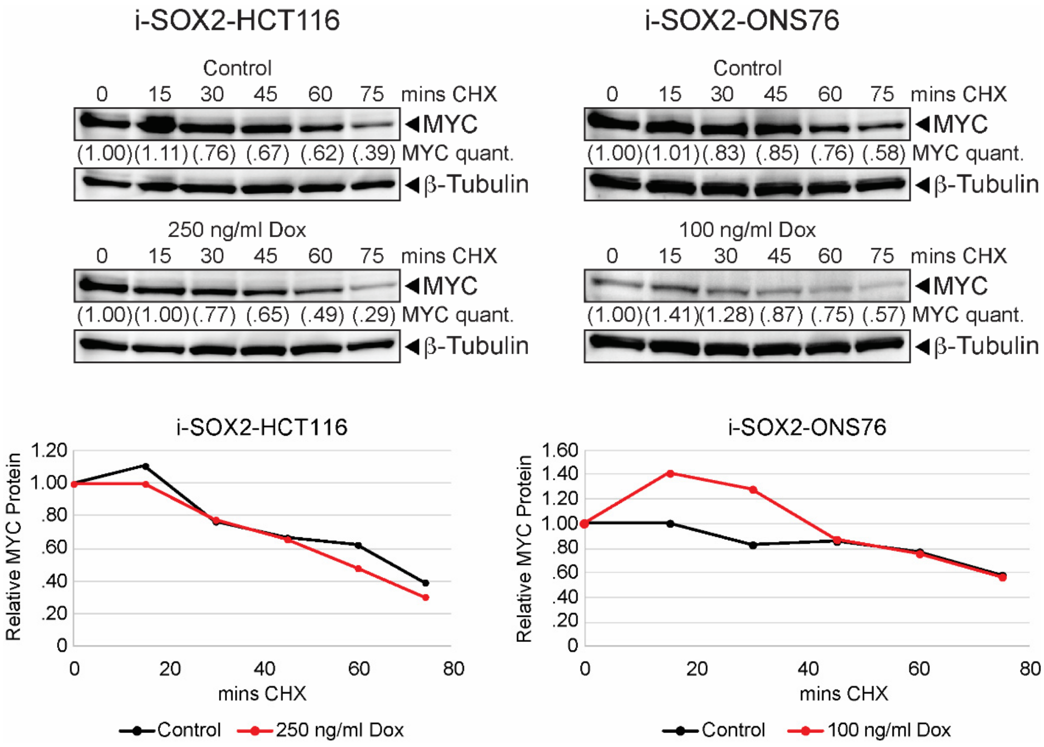

Figure S9. SOX2 elevation does not significantly affect MYC protein half-life. Western blot analysis of MYC protein levels in i-SOX2-HCT116 and i-SOX2-ONS76 cells after 24 hours treatment at the indicated Dox dosage followed by 50 mg/ml cycloheximide treatment for the times indicated. MYC protein levels were normalized to 0 cycloheximide time point.

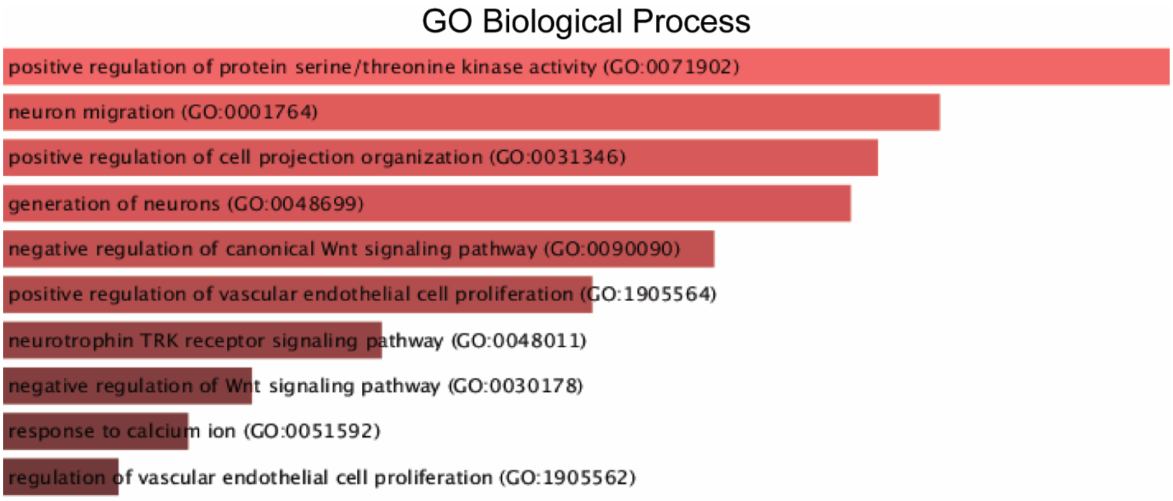

Figure S10. Gene ontology analysis of genes upregulated by elevated SOX2 in i-SOX2-ONS76 cells and i-SOX2-LNCaP cells. Top 10 GO terms for genes upregulated by elevating SOX2 in i-SOX2-ONS76 cells and i-SOX2-LNCaP cells.
